# Poor prognostic factors in patients who underwent surgery for acute non-occlusive ischemic colitis

**DOI:** 10.1186/s13017-015-0003-z

**Published:** 2015-03-08

**Authors:** Minsu Noh, Song Soo Yang, Seok Won Jung, Jae Ho Park, Yeong Cheol Im, Kyu Yeol Kim

**Affiliations:** Department of Surgery, Ulsan University Hospital, 290-3 Jeonha-dong, Dong-gu, Ulsan 682-714 Korea; Department of Digestive medicine, Ulsan University Hospital, Ulsan, Korea

**Keywords:** Ischemic colitis, Surgery, Postoperative mortality, Risk factors

## Abstract

**Introduction:**

Ischemic colitis (IC) is a disease with high postoperative morbidity and mortality. Knowledge of the risk factors for postoperative mortality could be helpful in clinical decision making and in optimizing postoperative treatment.

**Methods:**

From a prospective database, we conducted a retrospective medical record review of 50 patients who underwent surgery for IC between 2003 and 2011 at our institution. We analyzed the causes and potential risk factors for early mortality after surgery for IC.

**Results:**

The early postoperative mortality and morbidity rates were 30.0% (15/50) and 54% (27/50), respectively. The two most common causes of death were multi-organ failure (66.7%, 10/15) and fulminant septic shock (20.0%, 3/15). Univariate analysis showed that postoperative mortality was significantly associated with preoperative nephropathy, coronary artery disease, a previous history of cardiovascular surgery, an ASA score ≥ 4, surgical delay ≥ 3 days, preoperative hemodynamic instability, and use of pre- and intraoperative adrenergic vasopressors. In the multivariate analysis, a previous history of cardiovascular surgery (odds ratio [OR], 8.2; 95% confidence interval [CI], 1.2–56.5) and surgical delay ≥ 3 days (OR, 5.7; 95% CI, 1.2–27.9) were identified as independent risk factors for postoperative mortality.

**Conclusions:**

Because surgical delay is an avoidable determinant of early mortality, a high index of suspicion and early surgical intervention can increase survival. A routine postoperative evaluation for IC may be helpful in patients with a previous history of cardiovascular surgery.

## Introduction

Despite improvements in perioperative care, the postoperative mortality rate after surgery for ischemic colitis (IC) remains high, and has been reported to be more than 30% [1-4]. This excess mortality rate is highest during the immediate postoperative period, however, patients who survive the initial acute IC event can achieve long-term survival. The median long-term survival after the initial acute IC event is more than 40 months [2,4]. Previous studies, which aimed to develop a predictive risk score for postoperative mortality of IC, suggest that the excess mortality is due to the difficulty in reaching a timely diagnosis and the effects of pre-existing medical conditions [1,5]. Recently, Reissfelder et al. reported on the predictive risk score by looking at various perioperative variables (acute renal failure, nonocclusive ischemic colitis, subtotal/total colectomy, serum lactate > 2.5 mmol, and pre- and intraoperative administration of catecholamines) that were predictive of postoperative mortality in patients undergoing surgery for ischemic colitis [2]. Additionally Anthony et al. demonstrated the effectiveness of this scoring system in predicting the postoperative mortality of IC [4].

However, the risk factors included in the scoring system and the other well-known risk factors such as age, gender, and pre-existing comorbidities that contribute to the postoperative mortality of IC are unavoidable. Because there is lack of information on the avoidable risk factors, identifying them will allow researches and physicians to develop focused strategies aimed at reducing the risk and improving outcomes.

The aim of this study was to assess the incidence of morbidity and mortality in patients undergoing surgical treatment for IC at our institution. We also aimed to assess the causes and predictive factors of early mortality, and to determine whether a delay in surgery was associated with higher postoperative mortality of patient who undergo an operation for IC.

## Materials and methods

We retrospectively reviewed our prospectively collected database of patients with IC, and identified 50 consecutive patients who underwent abdominal surgery for histologically established IC between January 2003 and May 2011 at the Ulsan University Hospital. The Ulsan University Hospital Institutional Review Board approved this study. Patients who underwent surgery for occlusive colitis secondary to volvulus, herniation, trauma, or cancer were excluded. Patients who underwent reoperations for early postoperative complications and concomitant small bowel ischemia were also excluded.

The following patient characteristics were recorded: age, gender, smoking history, alcohol consumption, body mass index (BMI), the presence of significant comorbidities (i.e., diabetes mellitus, hypertension, coronary heart disease, respiratory disease, nephropathy, and liver cirrhosis), American Society of Anesthesiologists (ASA) classification, history of previous abdominal surgery and cardiovascular surgery, initial presenting symptoms, delay in surgical intervention, preoperative laboratory data (white blood count, c-reactive protein, serum urea, serum creatinine, and serum lactate), preoperative SIRS/sepsis, preoperative hypotension, preoperative use of adrenergic vasopressors, localization of colonic ischemia (i.e., right, transverse, left, entire colon), indications for surgical intervention (i.e., stenosis, gangrene, or failure to respond to medical treatment), emergency surgery, type of operation (i.e., segmental colonic resection, subtotal/total colectomy, or colostomy), presence of peritonitis at the time of surgery, intra-operative blood loss (measured by collecting blood in the suction minus the rinsing fluid), intra-operative blood transfusion, covering stoma, operative time, and intraoperative use of adrenergic vasopressors.

Surgical delay was defined as surgical intervention performed ≥ 3 days after the first clinical symptoms appeared (abdominal pain, hematochezia, diarrhea, peritoneal signs, radiographic findings of ileus or free air, unexplained leukocytosis, or lactic acidosis). Intra-operative blood loss was assessed using the anesthetic protocol. Intraoperative blood transfusion was defined as an allogeneic blood transfusion during the surgical procedure. Transfusion was performed at the discretion of the operating surgeon and anesthesiologist. Hemodynamically significant intraoperative blood loss or a hemoglobin level of 8 to 10 g/dL was used as a threshold for transfusion, depending on the patient’s comorbidities. In the present study, we defined hemodynamic instability as low blood pressure (<90 mmHg) requiring infusion without the use of vasopressors [6,7]. Sepsis was assessed in every patient who presented and was diagnosed if two or more of the following conditions were present: (1) temperature > 38°C or < 36°C; (2) heart rate > 90 beats per minute; (3) respiratory rate > 20 breaths per minute or PaCO2 < 32 mmHg; and (4) white blood cell count > 12,000/cu mm, or < 4,000/cu mm, or > 10% immature bands [8]. The use of vasopressors corresponded to the use of catecholamines (dopamine, dobutamine, dopexamine, noradrenaline, or adrenaline), and was administrated when patients had low blood pressure/heart rate due to decreased blood volume.

Surgical outcomes were determined on the basis of the postoperative follow-up. Postoperative mortality was defined as in-hospital death. Complications occurring within 30 days were classified as septic or non-septic. Septic complications included wound infections, persistent bowel ischemia, anastomotic leak, leakage of rectal stump, pneumonia, and septic shock. Anastomotic leak and leakage of rectal stump were confirmed by re-laparotomy or percutaneous drainage. Non-septic complications included intestinal obstruction, pulmonary edema and effusion, acute myocardial infarction, and acute renal failure.

The main analyses were based on comparing patients who experienced in-hospital postoperative mortality after surgery with those who did not. The chi-square test and Student’s *t*-test were used for univariate analyses of categorical and continuous variables. Multivariate logistic regression models were used to identify independent predictors of postoperative mortality. A receiver operating characteristic (ROC) analysis was performed for assessing discriminative accuracy in predicting in-hospital mortality. Factors with a *p* value < 0.05 in the univariate analysis were included in the multivariate analysis. Statistical significance was defined as a *p* value < 0.05. Statistical analysis was performed using SPSS 18.0 software (SPSS, Chicago, IL, USA).

## Results

### Patients and characteristics

A total of 50 patients underwent surgery at our institution during the study period. The study population included 26 males (52%) and 24 females (48%). The mean age of all patients was 68 ± 14 years, and the mean postoperative hospital stay was 30 ± 3 days. Indications for the operation were bowel infarction (42.0%), free perforation (39.0%), failure of medical treatment (20.0%) and hemorrhage (2.0%).

Demographic characteristics and comorbid conditions of patients at the time of the surgery are summarized in Table 1. Thirty-nine patients (78%) had comorbidities including comorbid cancer, liver cirrhosis, coronary artery disease, history of previous abdominal and vascular surgery, diabetes mellitus, nephropathy, respiratory disease, and hypertension. Hypertension was the most common pre-existing condition (46%), followed by diabetes mellitus, nephropathy, and coronary artery disease. History of previous surgery was present in 24 (48%) patients; 12 patients underwent abdominal surgery including gastrointestinal surgery and abdominal aortic aneurysm surgery, and 9 patients (18%) underwent major cardiovascular surgery including coronary artery bypass surgery, aortoiliac surgery, and peripheral vascular surgery.

**Table 1:** **Demographic characteristics and comorbid conditions in the study population**

	**No. of patients (n = 50)**	**Proportion (%)**
Age (years) ± SD	68.68 ± 14.14	-
Gender (male)	26	52.0
Smoking	20	40.0
Alcohol habit	16	32.0
Mean BMI (kg/m^2^) ± SD	22.62 ± 3.48	-
Comorbidities	39	78.0
History of cancer	9	18.0
Diabetes mellitus	14	28.0
Hypertension	23	46.0
Liver cirrhosis	5	10.0
Coronary artery disease	13	26.0
Atrial fibrillation	11	22.0
Nephropathy	14	28.0
Respiratory disease^a^	2	4.0
Previous history of surgery	24	48.0
Cardiovascular surgery	9	18.0
Abdominal surgery	12	24.0
Others	8	16.0

^a^Respiratory disease included chronic obstructive pulmonary disease, bronchial asthma.

SD, standard deviation; BMI, body mass index.

The main symptoms prompting patients to seek medical advice were abdominal pain (92%), followed by hematochezia (30%) and diarrhea (22%). During physical examination, the peritoneal sign was positive in 33 patients (66%), and 21 (42%) were hospitalized when the symptoms appeared. We also analyzed the surgical delay which was defined as mentioned above. The mean duration of surgical delay was 5 ± 9 days.

Seven (14%) patients were preoperatively classified with an ASA score of II, 8 (16%) had an ASA score III, 23 (46%) had an ASA score IV, and 12 (24%) had an ASA score V. Twenty-five (50%) patients showed signs of systemic inflammatory response syndrome (SIRS). Of the 27 (54%) patients with hemodynamic instability, 18 (66.7%) suffered from severe septic shock and required vasopressors.

Forty-four patients (88%) underwent emergency surgery, which was defined as surgery within the first 24 h after hospital admission or in the first 24 h after presentation to our emergency department. Eighteen patients (36%) were found to have a perforated colon, and the median operative time was 192 min (range, 110-465 min) with a median blood loss of 650 ml (range, 100-8000 ml). Twenty-six patients (52%) were given blood transfusions due to the indications as defined above. Ischemia involving the left colon was observed in 33 patients (66%), ischemia of the transverse colon was seen in 3 (6%), and ischemia of the right colon was seen 11 patients (22%). Three patients (6%) had ischemia involving the entire colon. A right hemicolectomy was performed in 8 patients (16%), a transverse colon segmentectomy was performed in 3 patients (6%), a left hemicolectomy was performed in 8 patients (16%), a sigmoid resection was performed in 18 patients (36%), and a subtotal/total colectomy was performed in 11 patients (22%). In 2 patients, a transverse colostomy was performed. A total of 34 patients (68%) received a stoma. Continuity of the bowel was not restored in any of the patients, so a Hartmann’s pouch was created. A total of 16 patients (32.0%) was performed an anastomosis, of which 13 patients (81.2%) were hemodynamically stable during surgery. We considered a second look surgery in patient who was suffered from septic shock and required vasopressors. In our study, six patients were planned a second look surgery. In these patients, four received planned surgery and two did not due to hemodynamic instability.

We also analyzed the pre- and intra-operative use of adrenergic vasopressors. A total of 19 patients (38%) received adrenergic vasopressor infusion due to septic shock intra-operatively. Nine (18%) of them had previously received adrenergic vasopressors at our institution the day before surgery.

Within 30 days of the procedure, 27 patients (54%) experienced postoperative complications (Table 2). Of the 27 patients who experienced postoperative complications, 22 patients (44%) experienced septic complications and 5 (10%) experienced non-septic complications. The three most common major life-threatening complications were septic shock (20%), persistent bowel ischemia (8%), and wound infection (8%).

**Table 2:** **Outcomes of surgery for ischemic colitis**

	**No. of patients (%)**		
**Type of complications**	**Number N = 27 (54.0)**	**Re-operation N = 11 (22.0)**	**Mortality N = 15 (30.0)**
Septic complications	22 (44.0)	11 (22.0)	14 (28.0)
Wound infection	4 (8.0)	2 (4.0)	0
Persistent ischemia	4 (8.0)	4 (8.0)	3 (6.0)
Anastomotic leakage	2 (4.0)	2 (4.0)	0
Leakage of rectal stump	1 (2.0)	1 (2.0)	1 (2.0)
Pneumonia	1 (2.0)	0 (0)	0 (0)
Septic shock	10 (20.0)	2 (4.0)	10 (20.0)
Non-septic complications	5 (10.0)	0 (0)	1 (2.0)
Intestinal obstruction	2 (4.0)	0	0
Acute myocardial infarction	2 (4.0)	0	1 (2.0)
Acute renal failure	1 (2.0)	0 (0)	0 (0)

Eleven patients (22.0%) who had septic complications required reoperation, and 6 (54.5%) of them died. Reoperation itself did not influence the in-hospital mortality rate (*p* = 0.143). Two out of ten patients with septic shock received surgical intervention due to clinical deterioration that required re-exploration. Four patients developed persistent bowel ischemia, and all of them required additional ischemic bowel resection. Two patients with anastomotic leakage and 1 patient with rectal stump leakage needed re-operation due to generalized peritonitis. Two patients required reoperation due to wound infection.

Postoperative mortality rate was high, with 15 patients (30.0%) dying during the postoperative period. The median survival until death in these patients was 16 days (range: 2-62 days).

The most common causes of early postoperative mortality were MOF in ten patients (66.7% and fulminant septic shock in three patients (20%). Fourteen of the 15 patients who died (93.3%) experienced septic complications. Of the ten patients (20.0%) who experienced septic shock, eight of them eventually died due to multiple organ failure and two died of fulminant septic shock, even though 2 underwent reoperation. Three patients (6%) with persistent bowel ischemia died due to MOF (n = 2) and fulminant septic shock (n = 1), and 1 patient with rectal stump leakage died due to pneumonia. Another reason for postoperative mortality was cardiac arrest due to acute myocardial infarction.

Thirty-five (70%) patients were discharged alive from the hospital, and the median time from surgery to discharge was 24 days (range: 9-127 days). Among these patients, those with postoperative complications had a significantly longer mean hospital stay (42 days vs. 28 days, p < 0.045).

**Figure 1:**
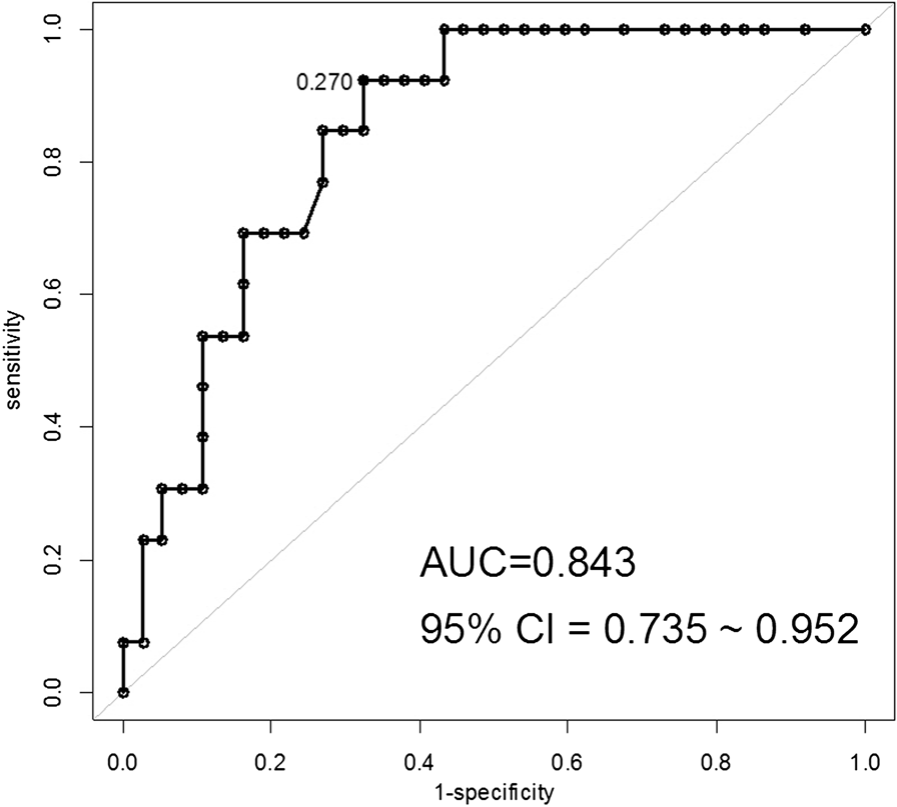
**Receiver operating characteristic curve for prediction of early postoperative mortality.**

Univariate analysis showed that postoperative mortality was significantly associated with preoperative nephropathy (*p* = 0.029), coronary artery disease (*p* = 0.023), a previous history of cardiovascular surgery (*p* = 0.006), an ASA score ≥ 4 (*p* = 0.039), surgical delay ≥ 3 days (*p* = 0.021), preoperative hemodynamic instability (*p* = 0.021), and severe hemodynamic instability requiring adrenergic vasopressors (*p* = 0.043). No other preoperative factors were associated with an increased risk of postoperative mortality, including patient age, gender, smoking history, hypertension, atrial fibrillation, respiratory disease, previous abdominal surgery, and localization of ischemia. The two groups did not differ in terms of clinical presentation, including abdominal pain, hematochezia, diarrhea, peritoneal sign, and preoperative SIRS. Moreover, intraoperative factors such as emergency surgery, type of surgery, bowel perforation, stoma formation, operative time, intraoperative blood loss, and blood transfusion were not associated with postoperative mortality (Table 3). In the multivariate analysis, a previous history of cardiovascular surgery (odds ratio [OR], 8.2; 95% confidence interval [CI], 1.2–56.5) and a surgical delay ≥ 3 days (OR, 5.7; 95% CI, 1.2–27.9) were identified as independent risk factors for postoperative mortality (Table 4). The rate of early postoperative mortality was 80.0% in patients who had both significant risk factors compared with the rate of early postoperative mortality of 8.7% in patients who had none of these risk factors (*p* = 0.003). These independent risk factors led to discriminative accuracy in predicting in-hospital mortality based on the area under the receiver operating characteristic curve of 0.825 (Figure 1).

**Table 3:** **Comparison of patient-related factors associated with postoperative mortality**

	**No. of patients (%)**		
	**Died (n = 15)**	**Survived (n = 35)**	***P*** **-value** ^**a**^
Mean age (years) ± SD	67.33 ± 12.87	69.26 ± 14.79	0.664
Gender			0.545
Male	9 (60.0)	17 (48.6)	
Female	6 (40.0)	18 (51.4)	
Smoking	7 (46.7)	13 (37.1)	0.547
Alcohol habit	3 (20.0)	13 (37.1)	0.328
Mean BMI (Kg/m^2^) ± SD	23.18 ± 2.86	22.39 ± 3.74	0.468
Liver cirrhosis	2 (13.3)	3 (8.6)	0.629
Nephropathy	8 (53.3)	6 (17.1)	**0.016**
Diabetes	4 (26.7)	10 (28.6)	1.000
Hypertension	7 (46.7)	16 (45.7)	1.000
Respiratory disease^b^	0 (0)	2 (5.7)	1.000
Coronary artery disease	7 (46.7)	6 (17.1)	**0.040**
Atrial fibrillation	4 (26.7)	7 (20.0)	0.713
Previous abdominal surgery^c^	6 (40.0)	6 (17.1)	0.146
Previous cardiovascular surgery^d^	6 (40.0)	3 (8.6)	**0.015**
ASA score (≥4)^e^	14 (93.3)	20 (57.1)	**0.019**
Localization of ischemia			0.445
Right colon	2 (13.3)	9 (25.7)	
Transverse colon	0 (0)	3 (8.6)	
Left colon	10 (66.7)	23 (60.0)	
Entire colon	1 (6.7)	2 (5.7)	
Preoperative initial symptoms			
Abdominal pain	14 (93.3)	32 (91.4)	1.000
Hematochezia	4 (26.7)	11 (31.4)	1.000
Diarrhea	3 (20.0)	8 (22.9)	1.000
Surgical delay (≥3 days)	11 (73.3)	12 (34.3)	**0.015**
Peritoneal irritation	11 (73.3)	22 (62.9)	0.533
Preoperative SIRS	10 (66.7)	15 (42.9)	0.217
Hemodynamic instability	12 (80.0)	15 (42.9)	**0.029**
Preoperative use of vasopressors	9 (60.0)	9 (25.7)	**0.028**
Preoperative laboratory data			
WBC (k/uL)	1315 ± 6.59	1421 ± 9.16	0.687
CRP (mg/dL)	13.56 ± 7.92	10.47 ± 11.62	0.353
Urea (mg/dL)	41.98 ± 25.01	26.08 ± 20.77	**0.024**
Creatinine (mg/dL)	4.64 ± 9.74	1.54 ± 1.30	0.068
Lactate (mmol/L)	2.84 ± 2.69	3.08 ± 2.88	0.791
Type of surgery			0.492
Rt. Hemicolectomy	1 (6.7)	7 (20.0)	
T-colon segmental resection	0 (0)	3 (8.6)	
Lt. hemicolectomy	3 (20.0)	5 (14.3)	
Sigmoid resection	7 (46.7)	11 (31.4)	
Subtotal/Total colectomy	4 (26.7)	7 (20.0)	
Colostomy	0 (0)	2 (5.7)	
Emergency surgery	14 (93.3)	29 (82.9)	0.659
Bowel perforation	6 (40.0)	12 (34.3)	0.754
Stoma formation	12 (80.0)	22 (62.9)	0.328
Operative time (min)	236.1 ± 86.0	213.0 ± 81.4	0.370
Estimated blood loss (>1000 mL)	7 (46.7)	10 (28.6)	0.329
Intraoperative blood transfusion	9 (60.0)	17 (48.6)	0.545
Intraoperative use of vasopressors	9 (60.0)	10 (28.6)	0.056

^a^Cross-table analysis using Fisher’s exact test.

^b^Respiratory disease included chronic obstructive pulmonary disease, bronchial asthma.

^c^Previous abdominal surgery included gastrointestinal surgery, abdominal aortic aneurysm surgery.

^d^Previous cardiovascular surgery included coronary artery bypass surgery, aortoiliac surgery, peripheral vascular surgery.

^e^ASA score according to the American Society of Anesthesiologists

BMI, body mass index; SIRS, systemic inflammatory response syndrome; SD, standard deviation

Bold print, P < 0.05.

**Table 4:** **Multivariate analysis of the risk factors associated with mortality**

	**Odds ratio**	**95% confidence interval**	***P*** **-value** ^**a**^
Gender, male	1.433	0.363-5.649	0.607
Age, ≥65 years	1.712	0.410-7.139	0.461
Nephropathy	3.002	0.559-16.118	0.200
Previous history of cardiovascular surgery	8.182	1.184-56.538	**0.033**
Surgical delay, ≥3 days	5.692	1.159-27.967	**0.032**
Preoperative hemodynamic instability	1.438	0.148-13.948	1.438
Preoperative use of vasopressors	1.071	0.126-9.067	0.950

^a^Cross-table analysis using binary logistic regression.

Bold print, P < 0.05.

## Discussion

In our retrospective study of 50 consecutive patients who underwent surgery for IC, 15 patients (30.0%) died within 30 days of surgery. Multi-organ failure and fulminant septic shock were the most common causes of early death, followed by cardiac arrest.

High rates of postoperative morbidity and mortality have been reported in patients with ischemic colitis who require surgery [1,2,9-12]. The mortality rates have been reported to range from 28% to 54%, and high morbidity rates ranging from 21% to 85% have been reported in patients with ischemic colitis requiring surgery [1-3,9,12,13].

In our study, a relatively lower postoperative mortality rate of 30% was demonstrated, which is in line with the published data. The overall postoperative complication rate of 54% in our study was also favorable when compared to the ranges reported in recent studies.

This study shows that sepsis and organ failure are common complications after surgery for IC. In almost all of the patients who died, the development of MOF and septic shock were significantly associated with postoperative mortality. These findings are in agreement with those in recent studies involving more than 100 patients who underwent surgery for IC, MOF and sepsis were found to be the principle causes of death [2,3]. Sepsis is a common postoperative complication in IC patients, and has been shown to be associated with postoperative mortality [4,14]. Our findings concur with those of previous studies, and we found that the development of postoperative sepsis is associated with up to a fifty-fold increase in mortality following surgery for IC.

In our study, septic complications, including wound infection, persistent bowel ischemia, anastomotic leakage, and septic shock were the most common postoperative complications. Septic shock was the most serious complication closely related to postoperative mortality, and occurred concurrently with or before MOF in most cases.

These findings highlight the observation that the development of MOF is strongly associated with sepsis. It has been postulated that progression to MOF exists in continuum with SIRS. Given that most of the patients with complications meet the criteria for SIRS, it is not surprising that MOF is common in IC patients.

Although the development of MOF does not predict mortality, a recent study found a greater than 50% prevalence of MOF among non-survivors of IC [2]. Two-thirds of postoperative mortality occurrences in our study were due to MOF.

The progression from the SIRS to MOF is not well explained, although some of the mechanisms responsible for it have been recognized. A surgery performed too late will favor MOF occurrence because of the inflammatory factors released by the necrotic intestine [15]. To prevent MOF, early surgical intervention and an aggressive approach may decrease the inflammation associated with IC.

Despite constant advances, IC patients’ postoperative mortality and morbidity rates remains high [16,17]. To achieve better outcomes, many studies have demonstrated which predictive factors are associated with postoperative mortality. We aimed to determine the predictors of early postoperative mortality of IC and found that two variables were independent predictors of 30-day mortality; surgical delay of 3 days or more and a previous history of cardiovascular surgery.

Several patient- and surgery-related characteristics have been previously found to be associated with in-hospital mortality in patients undergoing surgery for IC; preoperative lactate level, ASA classification, emergency surgery, acute renal failure, elevated intraoperative blood loss, allogeneic blood and fresh plasma transfusions, occlusion of the mesenteric artery, low output heart failure, catecholamine administration, and a previous history of cardiovascular surgery [1-4].

Recently, researchers have also attempted to validate a scoring system to predict postoperative mortality after surgery for IC [2]. In doing so, they identified that acute renal failure, serum lactate, intraoperative catecholamine, non-occlusive etiology, and total/subtotal colectomy are the components of the score. Although our findings are similar in some respects, that study only looked at a selective group of comorbidities and did not include surgical delay as a variable. We showed that a surgical delay of 3 days or more is a significant predictor of postoperative mortality. Our findings are supported by studies suggesting that early surgery is associated with lower mortality rates in patients requiring surgery for IC [2,17].

However, one of the main problems of ischemic colitis is the difficulty in reaching an adequate, early diagnosis. The clinical need for sensitive and specific non-invasive tests for detecting IC was highlighted in a recent review of the condition because its diagnosis still represents a challenge for physicians [18,19]. Absence of typical clinical findings because of old age, underlying disease, altered mental state, or medications has long been recognized as a reasons for the delay in surgical evaluation and intervention [20]. In such patients, the important issue is how to identify the more critically ill patients and make the most appropriate diagnostic and therapeutic decisions. In our study, more than two-thirds of deaths due to surgical delay occurred in patients who were bedridden, had an altered mental status, had no peritoneal signs, antecedent opioid analgesia, needed mechanical ventilation, and were in the postoperative state. Our study confirms the diagnostic difficulties and extremely poor prognosis in patients with underlying IC. High clinical suspicion, repeated abdominal exams, radiologic investigation, and most importantly, early surgical intervention, are recommended to reduce the significantly high mortality rates associated with surgical delay [21,22].

Additionally, we analyzed surgical delay in patients who experienced in-hospital mortality. When we compared the surgical delay between hospitalized and non-hospitalized patients, although there was no statistically significant difference, we found that the mean surgical delay was longer in the hospitalized group than in the non-hospitalized group (12 vs. 3 days, p = 0.397). These findings show that the physician’s suspicion of acute IC should not be restricted only to patients who are newly admitted to the emergency unit, but should also include patients currently in the hospital.

According to the previously published data, the incidence of IC varies from 1.2-4.6% after cardiovascular surgery [6,7,23-26]. Although the incidence is relatively low, IC has been recognized as a common determinant of mortality after cardiovascular surgery. This was also observed in our study, in which 6 of 9 patients (67%) who underwent cardiovascular surgery died due to IC. A previous retrospective study in a cohort of more than 600 patients who underwent aortic aneurysm repair showed that postoperative IC occurred in 4.1% patients within 2 days of surgery, with a subsequent mortality rate of 58.8%.

Postoperative diagnosis of IC should be systematically suspected after every cardiovascular surgery whenever digestive symptoms, unexplained sepsis, or hemodynamic instability occur [6]. As demonstrated in the Bjorck series, early passage of stools or hemodynamic instability are suggestive of postoperative IC [6]. These signs should be followed up by an endoscopic examination [27]. On the other hand, in one large study including almost 5000 patients, the mean time to diagnosis was 5.5 days following aortic surgery, regardless of the presentation [28]. Therefore, recommendations have been made for the routine performance of a postoperative colonoscopy, despite the absence of symptoms, as an in-hospital preventive strategy to reduce IC-related mortality [29,30].

This study had several limitations. First, because of small number of patients in our study, the mortality rate might not be representative of all patients who underwent surgery for IC and it could have relatively favorable outcomes in comparison. The limitation of small number of patients also affects out results in regards to the prognostic factors of patients who underwent surgery for IC, but we included almost all variables and identified parameters that had emerged in other studies [5,17]. Second, owing to its retrospective design, we could not be sure whether other factors may have influenced the postoperative outcomes, including intraoperative fecal spillage and tension on the anastomosis. Moreover, the surgeon’s assessment of the state of ischemic tissue at the time of surgery is important when deciding how much of the intestine should be resected and whether to perform an anastomosis. This assessment is subjective and difficult to quantify. However, all patients in this study were followed-up in the same unit by the same group of physicians, who used similar guidelines and made decisions collectively.

## Conclusion

Our study shows that the mortality and morbidity rates remain high after surgery for IC. The predominant causes of postoperative mortality after surgery for IC were MOF and fulminant septic shock. Patients at greatest risk of early postoperative mortality are those who have a previous history of cardiovascular surgery and a delay of 3 or more days from symptom onset to surgery. Because surgical delay is an avoidable determinant of early mortality, especially in hospitalized patients, a high index of suspicion and early surgical intervention can increase survival. Moreover, the correlation observed between a previous history of cardiovascular surgery and death from IC in the early postoperative period may provide the rationale for routine evaluation of IC in selected high-risk patients. A prospective study is needed to predict the risk of postoperative mortality in patients with ischemic colitis.

## Consent

Written informed consent was obtained from the patient for the publication of this report and any accompanying images.

## Abbreviations

IC: Ischemic colitis
BMI: Body mass index
ASA: American Society of Anesthesiologists
SIRS: Systemic inflammatory response syndrome
MOF: Multiple organ failure
CI: Confidence interval
OR: Odds ratio
SD: Standard deviation
