# Erratum to: Poor prognostic factors in patients who underwent surgery for acute non-occlusive ischemic colitis

**DOI:** 10.1186/s13017-016-0079-0

**Published:** 2016-06-02

**Authors:** Minsu Noh, Song Soo Yang, Seok Won Jung, Jae Ho Park, Yeong Cheol Im, Kyu Yeol Kim

**Affiliations:** Department of Surgery, Ulsan University and Ulsan University Hospital, 290-3 Jeonha-dong, Dong-gu, Ulsan, 682-714 Korea; Department of Digestive Medicine, Ulsan University and Ulsan University Hospital, Ulsan, Korea

## Erratum

Erratum to: World Journal of Emergency Surgery 2015, 10:12 doi: 10.1186/s13017-015-0003-z.

Following publication of the original version [1] of the article in World Journal of Emergency Surgery, it was brought to our attention that there was a mistake to the Author details section.

The author’s details should be presented as follows:

^1^Department of Surgery, Ulsan University and Ulsan University Hospital, 290-3 Jeonha-dong, Dong-gu, Ulsan 682-714, Korea.

^2^Department of Digestive medicine, Ulsan University and Ulsan University Hospital, Ulsan, Korea.

## References

[1] Noh (2015). Poor prognostic factors in patients who underwent surgery for acute non-occlusive ischemic colitis. World J Emerg Surg.

